# Predictors of Success of Different Treatment Modalities for Management of Ectopic Pregnancy

**DOI:** 10.1155/2014/423708

**Published:** 2014-12-14

**Authors:** Sümeyra Nergiz Avcioğlu, Sündüz Özlem Altinkaya, Mert Küçük, Selda Demircan Sezer, Hasan Yüksel

**Affiliations:** ^1^Department of Gynecology and Obstetrics, School of Medicine, Adnan Menderes University, 09010 Aydın, Turkey; ^2^Department of Gynecology and Obstetrics, School of Medicine, Muğla Sıtkı Koçman University, 48000 Muğla, Turkey

## Abstract

*Aim*. The purpose of this study was to investigate factors affecting the success of different treatment modalities for the management of ectopic pregnancy (EP). *Methods*. One hundred and ninety-seven patients with EP, were included in the study. Patients were treated with either intramuscular methotrexate (Mtx) or surgical treatment. *Results*. Mtx was applied in 97 (49.2%) patients. In 67 patients (69.1%), a single dose of Mtx and in 30 patients (30.9%) a multiple dose of Mtx was applied. Forty-seven (70.14%) patients were successfully treated with a single-dose Mtx. In the multiple-dose group, the success rate was 70% (21/30 patients). The difference between the success rates was not statistically significant. When the initial serum *β*hCG value was <1000 mIU/mL, the overall success rate of Mtx treatment was determined to be 86.11%; however, the rate decreased to 42.3% when the *β*hCG value was >3000 mIU/mL. On the other hand, if the EP mass diameter was <25 mm, the success rate was 89.28% and decreased to 52.63% when it was ≥25 mm. *Conclusion*. The results of the study showed that single-dose treatment with Mtx could be as successful as multiple doses. Overall success of Mtx treatment depended on initial *β*hCG value and EP mass diameter.

## 1. Introduction

Ectopic pregnancy (EP) is a potentially life-threatening condition and still the major cause of maternal mortality in the first trimester of pregnancy. It accounts for approximately 10% of maternal deaths [1]. In most developed countries, the incidence of EP has increased considerably over the last 20 years and now accounts for 1%-2% of all pregnancies [2–5]. Despite major advances, early diagnosis of EP is still a challenge for clinicians [1, 6, 7]. In the past 20 years, the use of sensitive *β*hCG tests, high-resolution transvaginal ultrasound, and advances in laparoscopy (LS) have enabled the detection of EP without tubal rupture. In the case of early detection, the possibility and success of noninvasive medical treatment as an alternative to surgical treatment increase [8].

EP has a significant detrimental effect on future fertility and less than half of the women who experience EP will be able to conceive again [5]. Thus, preserving the fertility of women has been the main goal of treatment in EP for gynecologists and over the last five years systemic methotrexate (Mtx) has been used for conservative treatment [9, 10]. In recent studies on single-dose Mtx therapy, tubal patency rates have been reported in excess of 80% and this is thought to be appropriate for preserving the fertility of patients [11].

However, there is still controversy about which patients will benefit from Mtx treatment. In the present research, we aimed to investigate the predictors related to the success of different treatment modalities for EP.

## 2. Materials and Methods

We enrolled patients with EP admitted to the Obstetrics and Gynecology Clinic at the Adnan Menderes University School of Medicine. The diagnosis was based on abnormally low *β*hCG doubling rates less than every 48 hours or plateauing levels with no evidence of an intrauterine pregnancy or sonographic identification of a gestational sac outside the uterus. Women presenting with acute or severe abdominal pain were immediately treated surgically. Those who did not require an immediate surgical intervention underwent repeated blood tests for *β*hCG levels and a complete blood cell count, as well as repeated transvaginal ultrasonographic examinations and measurements of blood pressure and pulse. Thus, women with EP in the first group were treated with surgical methods. All women diagnosed with unruptured EP undergoing the conservative treatment were included in the second group as a comparative method. Mtx was used for conservative treatment. Patients received 50 mg/m^2^ Mtx. The first injection was performed according to the protocol set down by Stovall and Ling [12]. If the *β*hCG level decreased by 15% or more between days 4 and 7, the treatment protocol was accepted as successful.

We measured *β*hCG levels every week until they were lower than 15 mIU/mL. We injected another Mtx dose when *β*hCG decreased by less than 15% or a plateau was reached in serum *β*hCG levels after Mtx treatment. Surgical intervention was performed in cases of tubal rupture and in patients whose *β*hCG levels decreased by less than 15% or a plateau was reached in serum *β*hCG after Mtx treatment doses. Tubal rupture was diagnosed on the basis of hemodynamic and clinical signs such as a rapid drop in blood pressure, increased abdominal pain, the presence of blood in the abdomen cavity that were confirmed with ultrasound, and a decrease in hemoglobin values. *β*hCG levels were measured using AxSYM Microparticle Enzyme Immunoassay (Abbot, Abbot Park, IL). The results are given in mIU/mL.

The medical data was collected from the patients' medical histories and the biochemical laboratory. Data including age, parity, gravity, diameter of EP mass, localization of EP, and type of surgical procedures were also collected. Each patient gave formal consent for Mtx therapy.

### 2.1. Statistical Analysis

Results are presented as mean ± SEM or percentile. Statistical analysis was conducted using the Fisher exact test, Student's *t* test, and chi-square as appropriate. The odds ratio (OR) of the main outcome was calculated with 95% CI. Data analysis was carried out using the Statistical Package for Social Science 18.0 (SPSS, Chicago, IL). *P* < 0.05 was statistically significant.

## 3. Results

In the present research, 197 women diagnosed as having EP were included. The mean age of women registered with EP was 30.61 ± 5.8 years (range 18–48). In 86 (43.7%) patients, an EP mass was observed in the right salpinx, in 65 (33%) in the left salpinx, and in 6 (3%) in the cornual region, and in 3 patients (1.5%) who were detected with ovarian, 3 patients (1.5%) with cervical, and 2 patients (1%) with caesarian scar ectopic pregnancy. In 32 patients (16.2%), no mass occurrence related to EP was determined. Rupture of EP was observed in 67 (34%) of patients. Clinical characteristics of rupture and nonrupture groups are presented in Table 1.

**Figure 1:**
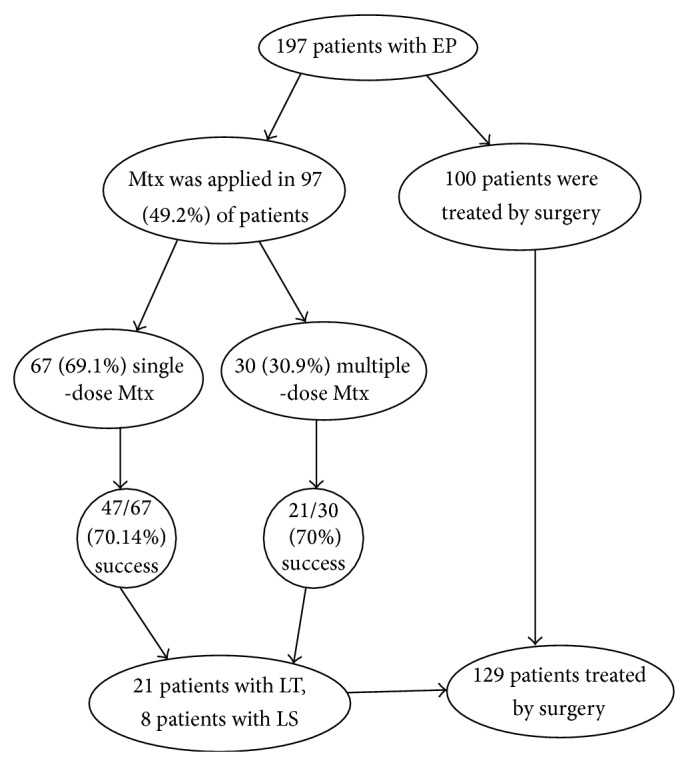
Flow of participants through the study.

In 97 (49.2%) patients LT was applied for EP; in 32 (16.2%) patients LS was applied for EP. In 68 (34.5%) patients, surgery was not performed. Mtx was applied in 97 (49.2%) patients. In 67 (69.1%) cases, a single-dose Mtx was applied and in 30 (30.9%) cases a double-dose Mtx was applied. 47 (70.14%) out of 67 patients were successfully treated with single-dose Mtx. In the double-dose group, the success rate was (21/30 patients) 70%. Among the patients treated with Mtx, 29 women required surgical management; 21 patients were treated with LT and 8 with LS. Only in 15 patients EP rupture was diagnosed intraoperatively. Of all participants, 129 patients were treated with surgery. A salpingectomy was performed in 96 (48.7%) patients; the operations were preserving tubal integrity like salpingostomy; partial salpingectomy or tubal milking was performed in 16 (8.1%), partial ovarian resection in 3 (1.5%), salpingoophorectomy in 9 (4.6%), and cornual resection in 5 (2.5%). In 68 (34.5%) patients, surgical procedure was not performed. The flow chart of the study is seen in Figure 1.

Based on the findings, patients receiving Mtx treatment were also divided into two different groups as follows: Group 1 (*n* = 67): patients with EP having a single-dose Mtx treatment; Group 2 (*n* = 30): patients with EP having a multiple-dose Mtx treatment (Table 2). The diameter of the EP mass (mean ± SEM) was significantly shorter in Group 1 (15.77 ± 2.18) compared to Group 2 (32.96 ± 3.21) (*P* < 0.01). The mean baseline (initial) level of *β*hCG was 2978.64 ± 597.59 in Group 1, which was significantly lower than in Group 2 (6610.56 ± 1431.62, *P* value = 0.024).

The overall success rate of Mtx was 70.10%. The demographic and clinical characteristics of patients affecting the success of Mtx treatment in EP are seen in Table 3.

## 4. Discussion

The present study evaluated the factors affecting the success rates of Mtx treatment in the management of EP in our clinic. It was determined that the overall success rate was 70.1%. There have been a great many published studies in the literature about Mtx success rates. In a review article published in 2003, the crude overall success rate in 1327 women was estimated as 88.8% (1181 of 1327). The success rate has been reported to be between 75% and 96% in properly selected patients [9]. The ratio in the present study was lower than the data in the literature. We think that this might be due to the time wasted in referral procedures. However, we have concluded that the medical treatment of EP is a practical treatment.

There have been studies in the literature comparing single- and multiple-dose Mtx treatments. Some of these concluded that multiple-dose Mtx treatment was more successful than a single dose [13] while some determined no difference [14, 15]. In our study, there was no difference in the success rates between the groups. However, important issues affecting the success rate of Mtx have been determined in the present study. First of all, while, in some series, an increase in the treatment failure group with an advanced maternal age ≥35 years was noted [16], in the present study, we did not find such an association. Eskandar demonstrated that when the initial serum *β*hCG value was greater than 2000 mIU/mL, the medical failure rate increased. Also, it was determined that an embryonic sac diameter greater than 3.4 cm should be closely monitored for treatment failure [17]. In addition, women with a pretreatment *β*hCG level of 3000–4000 mIU/mL have a greater probability for surgery or multiple-dose treatment [18]. Similar to these studies in the literature, in the present study, only the initial serum *β*hCG level and the diameter of the EP mass were the factors affecting the success of both single- and multiple-dose Mtx treatment. As the serum *β*hCG measurement was the mainstay of a rapid and early pregnancy diagnosis and an accepted biochemical marker for successful trophoblastic implantation [19], the predictors of success of Mtx in our study were low *β*hCG values and an adnexal mass diameter of less than 25 mm.

In the present study, among patients treated with Mtx, 29 women required surgical management; 21 patients were treated with LT and 8 with LS. Only in 15 patients EP rupture was diagnosed intraoperatively. Therefore, one important issue to emphasize is that clinicians must be careful in deciding on an operative treatment for patients having pain who are under Mtx treatment. This is because the pain after Mtx treatment could be due to tubal abortion or stretching of the tube by a hematoma. Fear of rupture misleads clinicians to operate early on unruptured ectopic pregnancies that would otherwise resolve with medical management [20]. Similarly, in the present study, differentiating “separation pain” due to tubal abortion from pain due to tubal rupture was difficult and led to early surgical interventions.

In the present study, patients with EP were treated with LS or LT as surgical treatment. The rate of LT (75.19%) was higher than the data in the literature. In a previous study [21, 22], the preferred laparoscopic method was applied only in 26% of patients, and in another 63% LT was performed. There have been many reasons for the increased ratio of LT in the present research. First of all, the laparoscopic surgical treatment was the selected method for patients who had a stable hemodynamic condition. The surgeon's own skills, of course, seemed to have an importance in the selection of surgical method. Lastly, similar to the literature [23], it was very difficult to perform LS when the Body Mass Index of the woman was >30 kg/m^2^ and in patients having previous abdominal surgery, heavy hemoperitoneum, or cornual pregnancy.

In the present study, a salpingectomy was performed in 74.41% of the patients. RCOG determined that salpingectomy was applied in 90%–95.8% of patients in different studies [21]. The ampullary region of the fallopian tube has been reported as the commonest site of EP [24–26]. Agdi and Tulandi [27] reported 93.1% tubal, 2.4% interstitial, 3.2% ovarian, and 1% cervical EP. In the present study, in 43.7% patients an EP mass was observed in the right salpinx, in the left salpinx in 33% patients, and in the cornual region in 3% patients, and 1.5% were seen to have ovarian, 1.5% cervical, and 1% caesarian scar EP masses. Hence, the localization of the EP masses in the present study was similar to the literature.

In the present study, EP rupture was observed in 67 (34%) of patients. This ratio was higher than in other published studies [28]. It is thought that the higher rates are due to the fact that our clinic is a third-stage or tertiary medical care center and obstetric and gynecology practitioners in the region are reluctant to perform operations due to medicolegal problems and refer most of the patients requiring surgical treatment to the larger facilities. Additionally, the probability of EP rupture correlated with increased serum *β*hCG levels and the findings were similar to the literature [29].

This study has some limitations. Firstly, as we have mentioned before, our center is a referral hospital and there is the issue of the time wasted during referrals from periphery facilities. This is a factor that may increase the risk of complications of EP such as rupture and failure of the systemic Mtx treatment due to increased *β*hCG levels and the increased diameter of the EP mass. Secondly, there were a limited number of participants included in the present study and therefore the results of the study should be confirmed by multicentered research covering a higher number of cases.

In conclusion, EP is a common and serious problem with both high morbidity and maternal mortality. We can diagnose EP before the clinical signs appear and this gives us the possibility of applying the medical treatment with fewer complications. What the present study brings to current literature is that it emphasizes that serum *β*hCG levels and ultrasonographic measurements of the EP diameter are vital for the assessment of rupture risk, deciding treatment modality, and also the success of conservative treatment. It was especially determined that Mtx treatment had higher success rates when *β*hCG values were lower and the diameter of the EP mass was smaller. The clinician should therefore recommend Mtx for all women who have a stable hemodynamic condition, an unruptured EP, and the desire to preserve fertility.

## Figures and Tables

**Table 1:** Clinical parameters of rupture and nonrupture groups in EP.

	Group 1 (*n* = 67) EP rupture (+) (SEM)^1^	Group 2 (*n* = 130) EP rupture (−) (SEM)^1^	*P* ^*^
Age (years)	31.46 ± 0.74	30.18 ± 0.49	0.158
Gravidity	2.94 ± 0.19	2.49 ± 0.13	0.060
Parity	1.20 ± 0.12	0.93 ± 0.8	0.073
Hemoglobin (g/dL)	10.53 ± 0.27	11.69 ± 0.12	**<0.01**
Hematocrit (%)	30.97 ± 0.80	34.67 ± 0.36	**<0.01**
Platelet (×10^9^/L)	264.31 ± 10.92	264.93 ± 6.09	0.961
White blood cell count (×10^9^/L)	10.79 ± 0.48	9.05 ± 0.28	**0.02**
Neutrophil %	73.07 ± 1.46	66.88 ± 0.99	**0.01**
Neutrophil count (×10^9^/L)	7.94 ± 0.51	6.16 ± 0.24	**0.02**
*β*hCG^2^ (0 day) (mIU/mL)			
<1000 (mIU/mL)	10/56 (17.85%)	46/56 (82.15%)	**0.02**
1000–3000 (mIU/mL)	24/74 (32.43%)	50/74 (67.57%)	
3000–10000 (mIU/mL)	17/39 (43.58%)	22/39 (56.15%)	
>10000 (mIU/mL)	16/28 (57.14%)	12/28 (42.86%)	
Diameter of EP^3^ mass (mm)	38.17 ± 1.83	25.44 ± 1.80	**<0.01**
Endometrial thickness (mm)	7.56 ± 0.45	6.58 ± 0.32	0.83

^1^SEM: standard error of mean, ^2^ *β*hCG: beta human chorionic gonadotropin, ^3^EP: ectopic pregnancy, ^*^statistical significance *P* < 0.05.

**Table 2:** Comparison of patient characteristics between single-dose and multiple-dose treatments with Mtx.

	Single-dose Mtx^1^ (*n* = 67)	Multiple-dose Mtx^1^ (*n* = 30)	*P* ^*^
Age (years)	31.02 ± 0.78	29.73 ± 0.72	0.227
Gravidity	2.67 ± 0.24	2.80 ± 0.19	0.685
Parity	0.94 ± 0.11	1.13 ± 0.19	0.401
*β*hCG^2^ (day 0) (mIU/mL)	2978.64 ± 597.59	6610.56 ± 1431.62	**0.024**
*β*hCG^2^ (1st day) (mIU/mL)	2533.28 ± 425.95	6336.46 ± 1333.94	**0.010**
*β*hCG^2^ (4th day) (mlU/mL)	2344.11 ± 459.71	6575.70 ± 1557.45	**0.013**
*β*hCG^2^ (7th day) (mIU/mL)	2255.87 ± 486.61	5615.10 ± 1485.46	**0.039**
Diameter of EP^3^ mass (mm)	15.77 ± 2.18	32.96 ± 3.21	**<0.01**
Endometrial thickness (mm)	6.77 ± 0.47	6.62 ± 0.76	0.871
Overall success rate (%)	47/67 (70.14%)	21/30 (70%)	0.988

^1^Mtx: methotrexate, ^2^ *β*hCG: beta human chorionic gonadotropin, ^3^EP: ectopic pregnancy, ^*^statistical significance *P* < 0.05.

**Table 3:** Demographic and clinical characteristics of all patients treated with Mtx in EP.

	Group 1 (n = 68) (success group) (SEM^1^-%)	Group 2 (n = 29) (failure group) (SEM^1^-%)	*P* ^*^
Age (years)	30.77 ± 0.70	30.27 ± 1.06	0.696
Gravidity	2.54 ± 0.16	3.10 ± 0.34	0.150
Parity	0.89 ± 0.10	1.24 ± 0.22	0.174
*β*hCG^2^ (day 0)			
<1000 (mIU/mL)	31 (86.11%)	5 (13.89%)	**<0.01**
1000–3000 (mIU/mL)	22 (84.61%)	4 (15.39%)	
3000–10000 (mIU/mL)	11 (42.30%)	15 (57.7%)	
>10000 (mIU/mL)	4 (36%)	5 (64%)	
*β*hCG^2^ (1st day) (mIU/mL)	2535.22 ± 486.19	6463.06 ± 1245.42	0.06
*β*hCG^2^ (4th day) (mIU/mL)	2187.70 ± 436.81	7434 ± 1668.68	0.05
*β*hCG^2^ (7th day) (mIU/mL)	1584.66 ± 298.92	2446.79 ± 1688.29	**0.01**
Diameter of EP^3^ mass (mm)			
<25 mm	50 (89.28%)	6 (10.72%)	**<0.01**
25–35 mm	10 (52.63%)	9 (47.37%)	
>35 mm	8 (36.36%)	14 (63.64%)	
Endometrial thickness (mm)	6.41 ± 0.48	7.45 ± 0.70	0.229

^1^SEM: standard error of mean, ^2^ *β*hCG: beta human chorionic gonadotropin, ^3^EP: ectopic pregnancy.

^*^Statistical significance *P* < 0.05.
